# Analysis of Freeze–Thaw Damage of Cement Mortars Doped with Polyethylene Glycol-Based Form Stable Phase Change Materials

**DOI:** 10.3390/ma17153731

**Published:** 2024-07-27

**Authors:** Antonella Sarcinella, Sandra Cunha, Nuno Reis, José Aguiar, Mariaenrica Frigione

**Affiliations:** 1 Innovation Engineering Department, University of Salento, Prov. le Lecce-Monteroni, 73100 Lecce, Italy; antonella.sarcinella@unisalento.it; 2 Centre for Territory, Environment and Construction (CTAC), Department of Civil Engineering, University of Minho, Campus de Azurém, 4800-058 Guimarães, Portugal; sandracunha@civil.uminho.pt (S.C.); pg46560@alunos.uminho.pt (N.R.); aguiar@civil.uminho.pt (J.A.)

**Keywords:** sustainable mortars, phase change materials (PCMs), polyethylene glycol (PEG), behavior at normal exposure conditions, behavior at freeze–thaw cycles, mortar durability

## Abstract

The development of construction materials with the integration of phase change materials (PCMs) has been a topic of wide interest in the scientific community, especially in recent years, due to its positive impact on temperature regulation inside buildings. However, little is known about the behavior of materials doped with PCMs when exposed to accidental or severe environments. Currently, a large area of the planet experiences seasonal freeze–thaw effects, which impact the durability and performance of construction materials. Accordingly, the main objective of this study was to evaluate the damage caused by cyclic freeze–thaw actions on the behavior of a cement mortar, including a PEG-based form-stable PCM. An experimental methodology was developed based on the physical and mechanical characterization of mortars under normal operating conditions and after being subjected to freeze–thaw cycles. The results indicated that, under normal exposure conditions, the incorporation of aggregate functionalized with PCM led to a decrease in the mortar’s water absorption capacity, compressive strength, and adhesion. However, its applicability has not been compromised. Exposure to freeze–thaw cycles caused a loss of mass in the specimens and a decrease in the compressive strength and adhesion capability of the mortar.

## 1. Introduction

Currently, the integration of phase change materials (PCMs) into construction elements is a rapidly expanding topic in the academic community, with high interest and impact on society. PCMs have been used in construction materials due to their temperature regulation capacity in buildings [1]. In recent years, a significant increase in energy consumption across the world has been registered [2]. In 2022, around 34% of global energy demand and 37% of carbon dioxide emissions were associated with buildings [3]. Considering the expansion of the construction industry and the growing concern with comfort parameters in buildings, it is expected that this contribution will remain high over the next few years [2,4,5]. Thus, the development of new construction materials, including PCMs, has significantly evolved. Presently, there are several construction materials integrated with PCM, including mortars [6,7,8,9,10,11,12], natural-based construction materials [13,14,15], sustainable concretes [16,17,18,19,20,21], and even innovative bricks [2,22,23], boards [24,25,26,27] and blocks [28,29]. However, the application of PCMs can be much wider: they have been proposed in photovoltaic panels to increase their electrical efficiency [30,31] and in carbon-based materials with the aim of increasing their thermal conductivity [32,33,34]. The integration of a PCM in construction materials can be carried out through various incorporation techniques, namely encapsulation (microencapsulation [6,7,8,10,12,15,18,24] and macroencapsulation [20,21,22,23,30,31]), direct incorporation [11,25], stabilization [9,16,17,19,32] and immersion [26,27]. Although several research studies have been carried out in recent years, it is clear that there is a lack of work related to the durability of these materials, especially regarding their exposure to adverse conditions.

The behavior of mortars and concretes facing freeze–thaw actions is a topic of superior interest since a large part of the planet suffers from the seasonal effects of freezing and thawing [35]. The physical transition from water to ice causes an increase in water pressure in the pores of mortars or cement mixtures, which can cause cracks in their structure [35,36,37]. Exposure to cyclic freeze–thaw actions accelerates the expansion of cracks in the mortar/concrete matrix, which in turn leads to their deterioration and a weakening of the mechanical capacity [35,38]. Accordingly, several studies have been carried out reporting the behavior of concretes and mortars subjected to freeze–thaw cycles [39,40,41,42,43,44,45]. Dong et al. [41] investigated the behavior of concrete with Aeolian sand lightweight aggregate subjected to a freeze–thaw cyclic test. The selection of Aeolian sand replacing natural sand was based on the observation that the damage due to freeze–thaw in concrete was, in this way, inhibited, with an optimal percentage of sand incorporation between 20% and 30%. Other authors also paid special attention to the possibility of improving the material’s performance with respect to this severe action, for instance, using aerating agents capable of increasing the space available in the mortar matrix for the expansion of the ice [43], the incorporation of micro and nanoparticles to decrease the porosity, avoiding the water freezing in the material matrix [44], or the incorporation of microfibers [45], nanotubes [46,47], nanosheets [48], and hydrophobic agents [49]. Rashidi et al. [47] used halloysite nanotubes to enhance the properties of cement mortar subjected to freeze–thaw cycles. These authors reported that, by incorporating 2 wt% of nanotubes, it is possible to notably mitigate the damage of frost action. Ji et al. [45] studied the mechanical properties of various fiber cementitious materials under freeze–thaw action as a function of the fiber content and type. Several types of fibers were studied, i.e., carbon fibers, PVA fibers, and glass fibers. The results obtained allowed us to verify that the mechanical characteristics of the cementitious composites with fiber incorporation appreciably increase after freeze–thaw with increasing fiber content, with carbon fibers presenting the best performance. Wang et al. [49] analyzed the behavior of mortars incorporating hydrophilic materials, observing that the mass loss of superhydrophobic mortar after 220 freeze–thaw cycles was lower with respect to ordinary mortar subjected to the same aging, proving the higher freeze–thaw resistance of the former.

While numerous studies have been conducted to examine and mitigate the freeze–thaw behavior of mortars, very few have explored the use of PCMs for this purpose. Cunha et al. [50] studied the behavior of mortars based on different binders (cement, air lime, hydraulic lime, and plaster) with the incorporation of PCM microcapsules, the latter displaying a transition temperature of 22.5 °C. The results showed that the incorporation of a PCM generally resulted in higher losses of the material during the freeze–thaw action, affecting mortars based on air lime and hydraulic lime more significantly. In a more recent paper, the same authors [51] studied the impact of the freeze–thaw cycles in cement mortars with the direct incorporation of a PCM with a temperature transition of 22 °C. They observed that the incorporation of a higher content of free PCM resulted in better behavior due to a lower specimen mass loss; however, total destruction of the specimens was observed after a high number of freeze–thaw cycles. Recently, Yu et al. [35] studied the incorporation of PCM in cementitious composites with the aim of improving their freeze–thaw performance. A PCM with a transition temperature of 5 °C was selected for this purpose, as this transition temperature is very close to the solidification temperature of water. The obtained results proved that the developed composites were able to offer excellent performance in terms of resistance to freeze–thaw aging, showing low mass loss with a higher PCM content while maintaining high compressive strength.

It is clear how freeze–thaw cycles can cause significant structural damage over time and how their cumulative effects significantly reduce the lifespan of mortar or concrete structures. Buildings and infrastructure exposed to such conditions will require more frequent maintenance and repairs, increasing the overall lifecycle costs and reducing sustainability. In general, it is known that PCMs can improve the thermal properties of concrete, reducing the number of freeze–thaw cycles experienced and increasing its service life [52,53]. However, there are only a limited number of studies addressing this issue. Thus, studying the impact of PCMs on this process helps in understanding whether they mitigate or exacerbate such damage, thereby informing better material design and usage guidelines. Furthermore, studying the interaction between PCMs and freeze–thaw cycles can lead to optimized formulations of PCM-enhanced mortars, balancing thermal benefits with structural integrity. This can result in materials that are not only energy-efficient but also highly durable and suitable for a wide range of climatic conditions.

In this manuscript, the authors aimed to study the durability to freeze–thaw aging of a cement-based mortar incorporating an eco-sustainable PEG-based PCM with a transition temperature of around 35 °C that demonstrated being able to control the indoor temperatures [54,55]. However, their behavior due to this type of severe action cannot be ignored. Thus, the behavior of the cement mortar containing PCM-based aggregates under freeze–thaw actions is compared to standard operating conditions (20 °C), evaluating the mass loss and mechanical behavior of these mortars. This work has an additional goal: the assessment of its practical applicability in real-world scenarios. By thoroughly understanding their behavior under various environmental conditions, including freeze–thaw cycles, researchers can provide more accurate recommendations and guidelines for their use in construction projects, ensuring safety, effectiveness, and longevity.

## 2. Experimental Design

### 2.1. Materials

The cement used to prepare the mortars was Portland cement CEM I 42.5 R, according to EN 197-1 [56], obtained from SECIL Company (Lisbon, Portugal). The chemical composition of the cement shows a loss of ignition value of 2.86%. Calcium Oxide (CaO) was the major chemical component with a value of 62.37%, followed by Silicon Dioxide (SiO_2_) with a value of 19.83%, Aluminum Oxide (Al_2_O_3_) with a value of 4.44%, and Iron Oxide (Fe_2_O_3_) with a value of 3.35%. Other compounds are also present, such as Magnesium Oxide (MgO), Potassium Oxide (K_2_O), Sodium Oxide (Na_2_O), Sulfates (SO_3_), and Chlorides (Cl^−^), but with lower contents. The cement has a density of 3030 kg/m^3^.

A superplasticizer was selected with the aim of reducing the water/binder (W/B) ratio necessary in the mortar formulation. A polycarboxylic ether polymer superplasticizer was chosen, compatible with all cements compliant with the EN 197-1 standard [56]. It has a density of 1050 kg/m^3^ and was supplied by BASF (Masterplan SKY 627, Porto, Portugal).

Polyethylene glycol 1000 (i.e., PEG1000) was employed as PCM. The commercial PEG1000 displays a transition temperature between 35 and 40 °C and a density of 1200 kg/m^3^ [57]. It was provided by Sigma-Aldrich (Darmstadt, Germany). The choice of PEG1000 as the PCM was determined by several important factors. PEG1000 offers good latent heat capacity, which allows for effective thermal energy storage during phase transitions, ensuring long-term thermal stability. Furthermore, its compatibility with building materials proves its suitability for incorporation into various building elements. Finally, the choice of PEG1000 is in line with our goal of developing sustainable PCMs due to the non-toxic nature of this polymer and its environmental benefits [57].

The aggregate employed to include PEG1000 polymer in mortars was Lecce stone (LS), a calcareous stone material historically used in construction in Salento, Italy. The stone matrix used to produce the PEG-based PCM was obtained from waste from stone cutting in a quarry located near the city of Lecce. It was supplied by a local company (L’essenza della pietra, Melissano, Lecce, Italy). Taking into account its origin, it was necessary to apply a grinding treatment (using a mill) to the stone waste. Subsequently, through a sieving operation, particles with dimensions between 1.6 mm and 2.0 mm were obtained. The bulk density of the stone aggregate was 2957 kg/m^3^. Regarding the chemical composition, the main constituent of LS is calcium carbonate (CaCO_3_), with a content between 92 and 95%. This stone has a high porosity, making it an ideal candidate for PCM production. The inclusion of PEG1000-PCM into the LS aggregate was carried out under vacuum at 60 °C employing the well-known “form-stable method”; in this way, the PEG in its liquid state was forced to penetrate the pores of the LS aggregate [57].

### 2.2. Mortars Design

**Figure 1:**
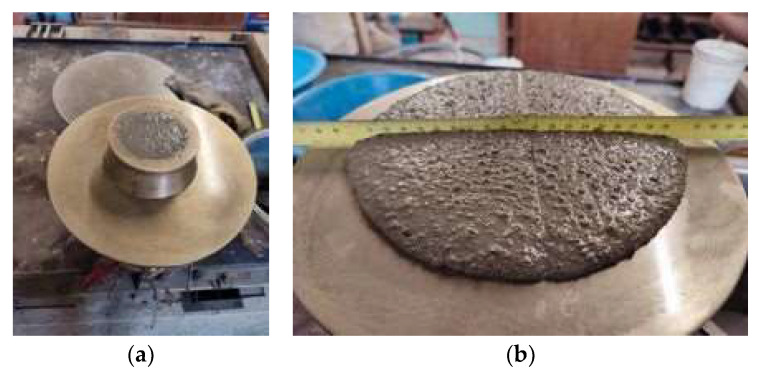
Workability test: (**a**) Truncated conical mold with mortar; (**b**) spreading diameter.

The mortars were designed in order to obtain an area of 1 m^3^, maintaining a flow table workability of 170 ± 10 mm, according to the European specification EN 1015-3 [58] (Figure 1). A reference mortar, i.e., CEM_REF, was first developed using unfilled LS granules as aggregate. A second mortar was developed containing the LS aggregate impregnated by PCM, which is indicated as CEM_PCM mortar. The mortars were manufactured using a cement dosage of 1000 kg/m^3^ and a superplasticizer dosage of 2% with respect to the binder mass. However, taking into account the desired consistency of the mortars and the influence on the workability of the PEG–PCM aggregate, the water/binder ratio needed to be adapted. As a consequence, the W/B of the CEM_REF mortar was set at 0.39, and the water/binder ratio of the CEM_PCM mortar was 0.30. The determination of the optimal W/B ratio for each mortar was determined experimentally through multiple iterations. Table 1 presents the formulation of the developed mortars.

### 2.3. Methodology

**Figure 2:**
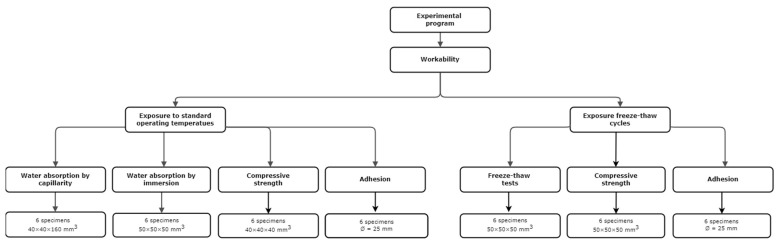
Experimental program diagram.

The experimental program aimed at investigating the effect of the aggregate containing PEG–PCM on the physical and mechanical properties of the cement mortar exposed to standard operating conditions (i.e., 20 °C and 60% R.H.) and after freeze–thaw cycles, with the mortar without PCM as a comparison (Figure 2). The experimental program involved twenty-four mortar specimens for investigations in standard operating conditions and twelve specimens for the study of behavior following exposure to freeze–thaw cycles.

Regarding standard exposure conditions, water absorption by capillarity and immersion tests, flexural and compressive tests, and adhesion tests were carried out on both mortar compositions. As regards the behavior of the mortars exposed to freeze–thaw cycles, compressive strength and adhesion tests were carried out.

The test of water absorption by capillarity was performed according to the European standard EN 1015-18 [59]. The same code was used for the determination of the coefficient of water absorption by capillarity. Three specimens were produced for each mortar (i.e., CEM REF and CEM PCM) with dimensions of 40 × 40 × 160 mm^3^. The specimens were cut to obtain the resulting halves (≈40 × 40 × 80 mm^3^). These samples were placed in an oven at a temperature of 60 °C until they reached constant mass (dry state). Then, the specimens were waterproofed on the sides, limiting water contact to one specimen face. Mass control measurements were carried out to quantify the amount of water absorbed by capillarity over a period of 29 days. In the first 5 h of testing, measurements were taken at 10, 30, 60, 90, 120, 180, 240, and 300 min. Throughout the test, a constant water height of 5 mm was maintained.

The test of water absorption by immersion was performed according to the Portuguese specification LNEC E 394 [60]. For each mortar, three samples with dimensions of 50 × 50 × 50 mm^3^ were prepared. Considering the need to adapt the mentioned code to mortars, the test began with the determination of the dry mass of the specimens, which was obtained after placing the specimens in an oven at 60 °C until a constant mass was reached. Subsequently, the samples were saturated with water until they reached their constant mass. Finally, their hydrostatic mass was determined.

**Figure 3:**
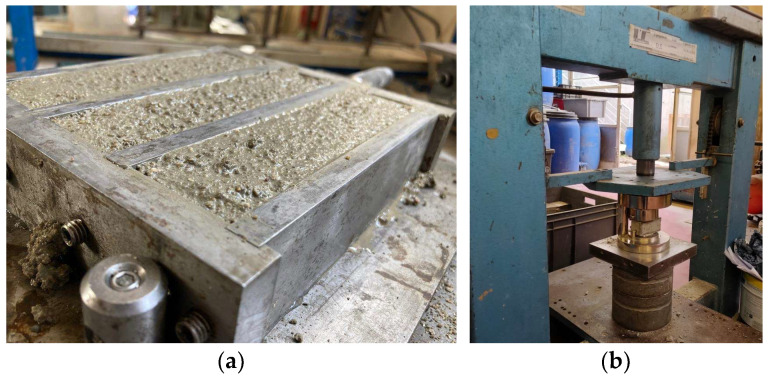
Mortar compressive strength: (**a**) Specimens molding; (**b**) compressive strength test.

The compressive strength tests were developed according to the European standard EN 1015-11 [61] (Figure 3). Three specimens of each composition were used, with dimensions of 40 × 40 × 160 mm^3^. The tests were carried out on an ELE AutoTest hydraulic press (ELE International, Milton Keynes, UK) with a test speed of 150 N/s. The compressive strength was determined based on the maximum load and the specimen’s loading area (40 × 40 mm^2^).

The adhesion tests were performed according to the European standard EN 1015-12 [62]. In this way, it was possible to estimate the adhesion of mortars at 28 days of age when applied to a ceramic substrate frequently used in the construction sector for masonry. The mortar was applied to one side of a brick. After its hardening, circular cuts measuring 50 mm in diameter were made on the mortar/brick system. Subsequently, metallic pieces with a circular base measuring 50 mm in diameter were glued to the test area. The test was then performed adequately, leveling the sample and exerting sufficient force to fracture it. The adhesion was determined by the maximum load and the specimen’s loading area.

**Figure 4:**
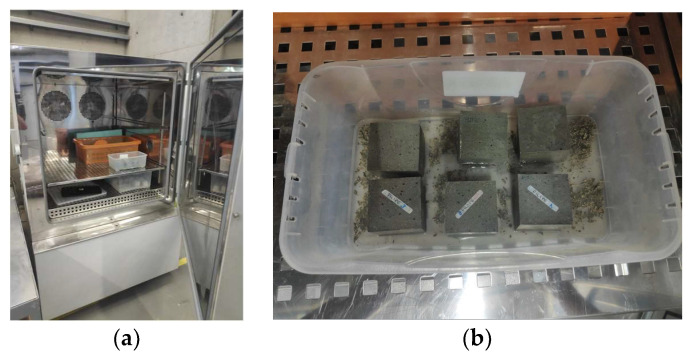
Freeze–thaw tests: (**a**) Climatic chamber; (**b**) specimens during the tests.

**Figure 5:**
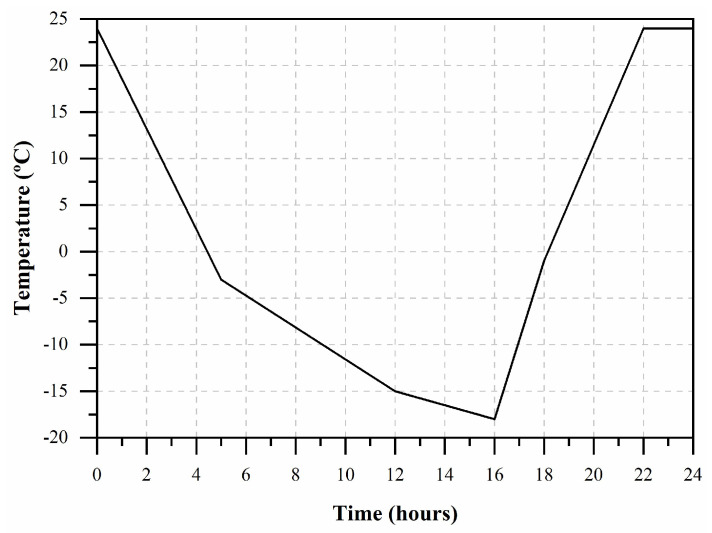
Temperature law used in freeze–thaw tests.

The freeze–thaw tests were carried out according to the European standard CEN/TS 12390-9 [63] to assess the behavior of mortars when subjected to this kind of aging, using a climatic chamber model Fitoclima 6400 EC25 N/S 1449 from ARALAB (Figure 4). The specimen’s degradation was recorded by measuring the loss of mass during 56 cycles of this test; the weight measurements were carried out before the first freeze–thaw cycle (cycle 0) and during the 56 test cycles, according to the program reported in Table 2. After 56 freeze–thaw cycles, compressive strength and adhesion were also determined for both mortar compositions. Each freeze–thaw cycle had a duration of 24 h, with the temperature varying between 24 °C and −18 °C, according to the scheme illustrated in Figure 5. Before carrying out the tests, the specimens were saturated. During the mortar production, high relative humidity was guaranteed. Likewise, during the freeze–thaw cycles, the contact of the specimens with a layer of water was guaranteed, ensuring that the specimens avoided any loss of mass due to water evaporation caused by the refrigeration system of the climate chamber. Three samples with dimensions of 50 × 50 × 50 mm^3^ were used in the freeze–thaw tests to monitor the mass of the specimens and determine the compressive strength. Tests of compressive strength were again performed according to the European standard EN 1015-11 [61], with the same procedure previously described. Regarding the adhesion tests, three cylindrical specimens with a diameter of 50 mm and a height of 10 mm were prepared, according to the European standard EN 1015-12 [62], following the procedure already described.

## 3. Results of Tests Performed on Mortars Kept in Standard Conditions

### 3.1. Water Absorbed by Capillarity

**Figure 6:**
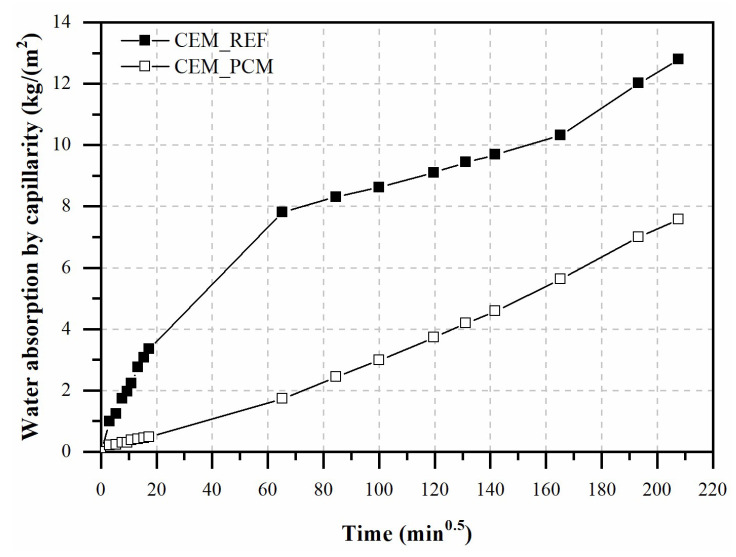
Water is absorbed by capillarity in both mortars, i.e., CEM_REF and CEM_PCM.

Figure 6 shows the water absorption capacity by capillarity of the mortars over the test period, i.e., 29 days.

The replacement of 100% of LS aggregate by LS flakes impregnated by PCM, in fact, resulted in a decrease in water absorbed by capillarity equal to approximately 39% compared to the CEM_REF mortar. 

**Figure 7:**
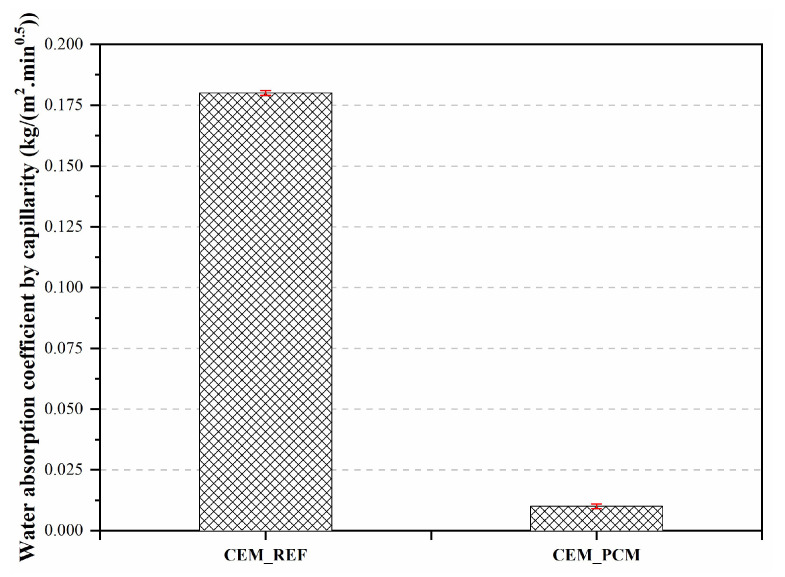
Coefficient of water absorbed by capillarity by both mortars, i.e., CEM_REF and CEM_PCM.

The coefficients of water absorption by capillarity, calculated for both mortars from the results of the test of water absorption, are reported in Figure 7. 

It was again possible to observe an appreciable decrease in the water absorption coefficient, down to 90%, for the mortar containing the PEG-based PCM compared to the CEM_REF one. 

The mortars under study, whether containing the PEG-based PCM or not, can be classified based on their coefficient of water absorption by capillarity according to the Portuguese specification NP EN 998-1 [64]. CEM_REF and CEM_PCM can both be included in the W2 class since a coefficient lower than 0.2 kg/m^2^.min^0.5^ was calculated for both.

### 3.2. Water Absorbed by Immersion

**Figure 8:**
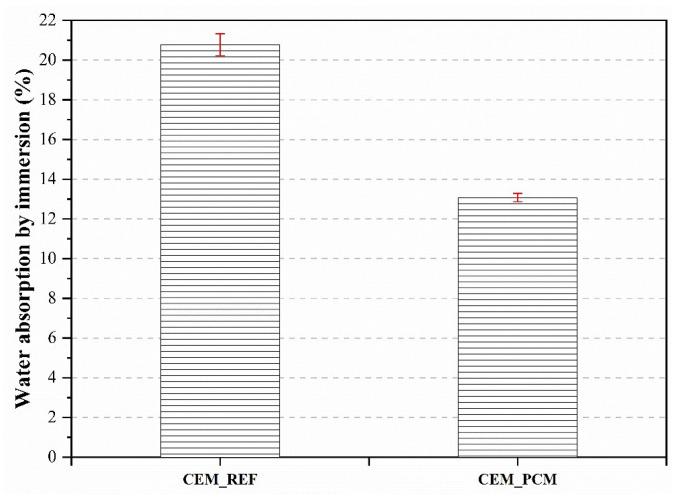
Water is absorbed by immersion in both mortars, i.e., CEM_REF and CEM_PCM.

The results obtained from the water absorption by total immersion test carried out on both mortars are illustrated in Figure 8. The mortar incorporating the PEG–PCM aggregate showed a lower water absorption capacity (by around 37%) than CEM_REF, even in the total immersion test.

### 3.3. Compressive Strength

**Figure 9:**
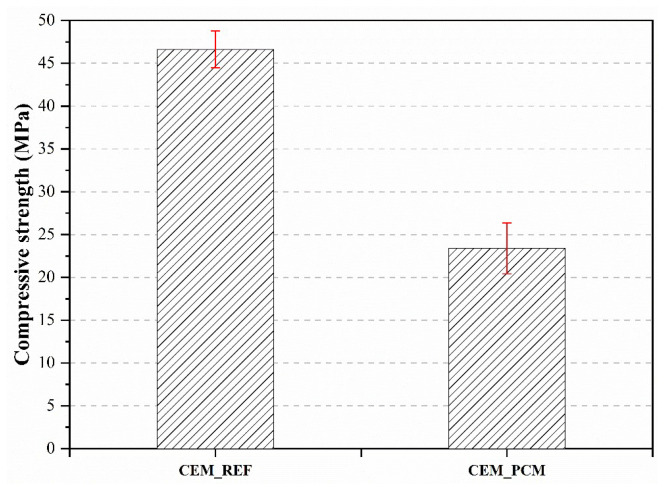
Compressive strength was measured on both mortars, i.e., CEM_REF and CEM_PCM.

The results, in terms of compressive strength values, of the mechanical tests performed on the produced mortars are reported in Figure 9. The addition of aggregates impregnated with PEG-based PCM to the cement mortar resulted in a decrease in the compressive strength of approximately 50% compared to the reference mortar. 

Although the developed mortar containing the PEG-based PCM displayed a decrease in compressive strength if compared to CEM_REF, it is important to highlight that plaster mortars do not have a structural function. Therefore, it is essential to verify whether or not the decrease in compressive strength of the CEM_PCM mortar could represent a limit for the application of this material as plaster in buildings. According to the NP EN 998-1 standard [64], plastering mortars are classified into four groups based on their compressive strength after 28 days of curing, namely CS I, CS II, CS III, and CS IV. Class CS I refers to mortars with a lower compressive strength, and class CS IV refers to mortars with the highest values of compressive strength. The mortars developed in this study, whether they contain PCM or not, fall into the CS IV category; therefore, there are no obstacles to their application in buildings.

### 3.4. Adhesion

**Figure 10:**
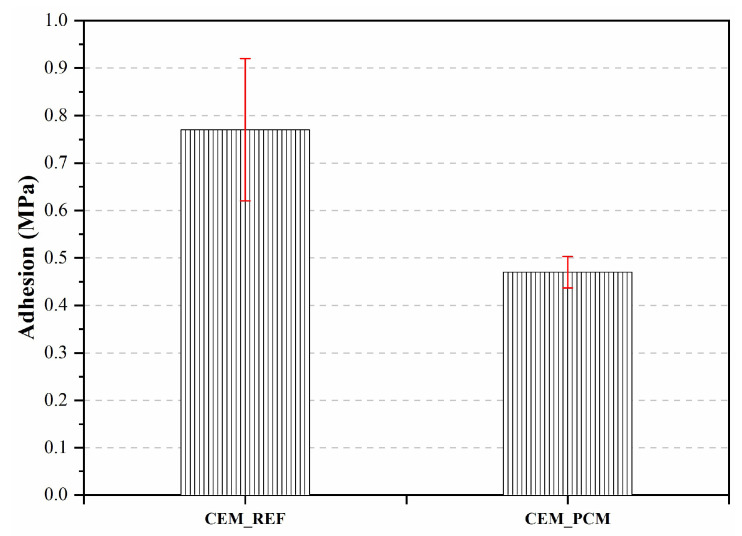
Adhesion strength was measured on both mortars, i.e., CEM_REF and CEM_PCM.

Figure 10 illustrates the results of the adhesion test carried out on both mortars with a ceramic support. A decrease in the adhesion strength of approximately 39% of the CEM_PCM mortar compared to the reference mortar (i.e., CEM_REF) was measured. 

## 4. Results of Tests Performed on Mortars Subjected to Freeze–Thaw Tests

### 4.1. Mass Loss

The mass loss measured on mortars is linked to their degradation due to freeze–thaw cycles. During the freezing cycle (at temperatures below 0 °C), the water contained in the pores of the mortar passes to the solid state, increasing its volume by approximately 9% [47]. The increase in the volume of water contained in the pores causes tension within the mortar matrix, which causes microcracks. Cyclic freeze–thaw processes cause the development of increasing internal tensions, with a consequent increase in microcracks, negatively impacting the durability of the mortar. The microcracks that develop during these cycles lead, in fact, to the loss of surface material in the samples, resulting in their partial or total destruction in the most severe cases.

**Figure 11:**
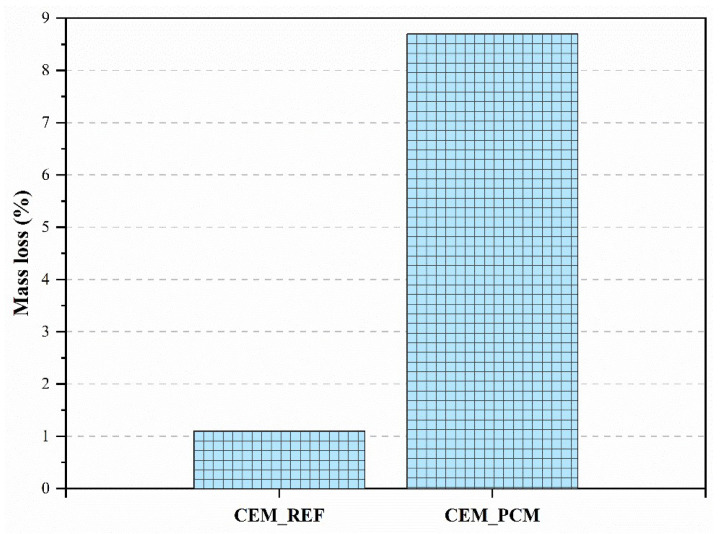
Mass loss by both mortars, i.e., CEM_REF and CEM_PCM, after 56 freeze–thaw cycles.

According to the results presented in Figure 11, the mortars incorporating PEG–PCM aggregates show a greater mass loss after 56 freeze–thaw cycles compared to reference mortars.

### 4.2. Compressive Strength

**Figure 12:**
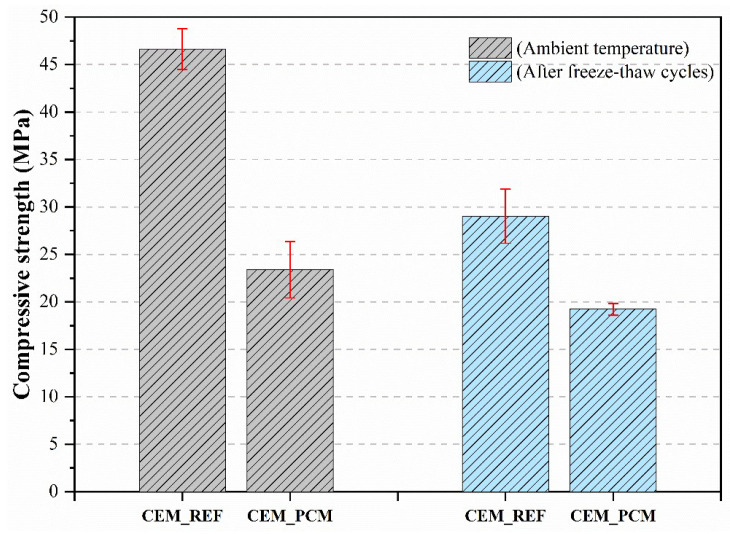
Compressive strength was measured on both mortars, i.e., CEM_REF and CEM_PCM, before and after 56 freeze–thaw cycles.

After subjecting the mortars to freeze–thaw cycles, their compressive strength was determined to evaluate their degradation following this aging. Figure 12 shows the results obtained: A decrease in compressive strength after the freeze–thaw cycles was recorded regardless of the composition of the mortars.

This behavior is due to the development of microcracks in the mortar caused by the action of the freezing and thawing cycles of water, as previously demonstrated by the mass loss of the mortars (Figure 11). 

Analyzing the individual mortars, the reference mortar shows a decrease in compressive strength of approximately 38% compared to the initial standard conditions. On the other hand, the compressive strength of CEM_PCM mortar, i.e., the composition that showed a more significant mass loss (as visible in Figure 11), was reduced by 18% following freeze and thaw cycles.

### 4.3. Adhesion 

**Figure 13:**
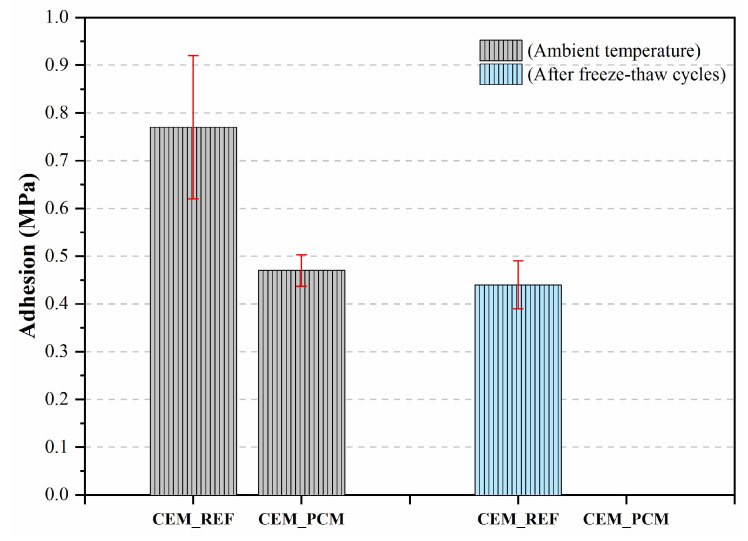
Adhesion strength was measured on both mortars, i.e., CEM_REF and CEM_PCM, before and after 56 freeze–thaw cycles.

In Figure 13, the results of the adhesion tests performed on both mortars under analysis before and after the freeze–thaw cycles are reported. For the reference mortar, a decrease in adhesion of approximately 43% was observed after exposure to freeze–thaw cycles compared to standard conditions. On the other hand, after exposure to freeze–thaw cycles, the mortars containing the aggregates impregnated with PCM showed very brittle behavior, and it was not possible to measure their adhesive strength. 

## 5. Discussion

The experimental program was designed to examine the impact of PEG–PCM aggregate on the physical and mechanical properties of cement mortar under standard conditions (20 °C and 60% R.H.) and after freeze–thaw cycles, using mortar without PCM as a control. For standard conditions, the tests conducted included water absorption by capillarity and immersion, compressive strength, and adhesion. For the mortars exposed to freeze–thaw cycles, compressive strength and adhesion tests were performed.

Starting from the standard conditions, it was possible to observe that mortar containing the PEG–PCM aggregate exhibited an appreciable decrease in the amount of water absorbed by capillarity if compared to the reference mortar. This behavior can be explained by the ability of PEG to reduce the porosity of the LS aggregate, which is essentially saturated by this polymer. Another factor that can also contribute to the reduced water absorbed is the presence of a lower water/cement ratio in the mortar incorporating the PEG–PCM aggregate, obtaining a more compact mortar matrix and, consequently, a lower porosity. Accordingly, the coefficients of water absorption by capillarity are lower for the mortar that contains the PEG-based PCM, confirming its lower porosity due to a high content of binder and a low water/binder ratio. A higher coefficient of water absorbed by capillarity was measured for the reference mortar; as already underlined, this is due to the greater absorption capacity of the natural porous aggregate, i.e., that not saturated by the PCM, and also to the greater water content used to make the reference mortar compared to CEM_PCM. Similar results were also reported by Dora and Mini [65] when evaluating the coefficient of water absorbed by the capillarity of cement mortars incorporating capric acid and ethyl alcohol (used both as PCMs) into expanded vermiculite (light aggregate) using the form-stable technique. Cunha et al. [66] demonstrated that the direct incorporation of PCM in cement mortars caused a decrease in water absorption by capillarity due to the total or partial filling of the mortar pores by the PCM. Other authors, although using a different incorporation technique (i.e., microencapsulation), have also reported the decrease in the water absorption coefficient by capillarity of cement mortars; the phenomenon was again justified by the filling of the porosities of the mortars by the PCM microcapsules [6].

Even in the total immersion test, the mortar incorporating the PEG–PCM aggregate showed a lower water absorption capacity than CEM_REF. This behavior can be again explained by the lower water content of the CEM_PCM mortar, leading to the formation of fewer pores in its matrix. Additionally, the LS aggregates in the CEM_PCM mortar are completely saturated by the PCM, significantly reducing its water absorption capacity. It can be concluded that the presence of the PCM creates an effective barrier against the penetration of water into the mortar, reducing its open porosity. This effect is also justified by the non-porous structure of the PEG-based PCM [65]; therefore, its incorporation leads to mortars with lower porosity than mortars without PCM [7].

This ability of the PCM-containing mortar to absorb less water both by capillarity and immersion offers several advantages from an application perspective, especially for those environments where moisture resistance is crucial. For instance, the lower water absorption makes the PCM mortar ideal for exterior plastering and coatings, protecting buildings from moisture ingress and related damage. Additionally, using PCM mortar in basements and foundation walls can help prevent water seepage and reduce the risk of mold and structural damage caused by moisture. The mortar’s resistance to water absorption also makes it a suitable choice for use in wet rooms, such as bathrooms, and other areas with high humidity, helping to maintain structural integrity and reduce maintenance needs. Furthermore, PCM mortar can be used in thermal insulation layers where both moisture control and thermal regulation are essential, such as in walls and roofs, improving energy efficiency while protecting against water damage. Lastly, in ventilated facade systems, PCM mortar can act as a protective and insulating layer, offering both thermal benefits and resistance to weathering and moisture. These applications take advantage of the low water absorption properties of the PCM-enhanced mortar, providing durability and efficiency in various construction scenarios.

Regarding the mechanical properties performed in compressive mode, the incorporation of PEG-based PCM-impregnated aggregates into the cement mortar led to an approximate 50% reduction in compressive strength compared to the reference mortar. As reported in several studies [8,51], this behavior is due to the presence of PCM in the microstructure of the CEM_PCM mortar, which is capable of delaying the hydration process of the cement. In addition, the accumulation of PEG-based PCM on the surface of the LS aggregate particles can reduce the adhesion between the binder paste and the aggregate, contributing to a reduction in the compressive strength of the CEM_PCM mortar [57,67].

Several authors have found a worsening of the mechanical behavior, in particular of the compressive strength, of mortars that incorporate shape-stabilized PCMs as aggregates [9,68]. A decrease in compressive strength has also been reported for mortars containing microencapsulated PCMs [7,8,69] or PCMs directly incorporated in the mortars [51,66]. Despite the drastic reduction in mechanical properties of the PCM-containing mortar compared to the reference mortar, it is important to note that the results classify it as a CS IV mortar according to the NP EN 998-1 standard [64]. Furthermore, since it is not intended to be a structural mortar but rather a plaster, the mechanical properties it has demonstrated are sufficient for its intended application.

Similarly, when studying the adhesion of these materials, a decrease in this property was observed for the CEM_PCM mortar. As already mentioned in the case of decreasing compressive strength, the reduction in adhesion is probably related to the delay in the hydration process of the cement in the mortar containing the aggregate impregnated by PCM [8,51,57,67]. The possible accumulation of PEG-based PCM on the surface of the LS aggregates may also have had a negative impact on the adhesion of the aggregate particles to the cement paste. This behavior has already been observed by the same authors in a previous study relating to the incorporation of microencapsulated PCM into mortars [70].

On the other hand, the results obtained from the mortars with and without PCM subjected to freeze–thaw cycles showed a significant mass loss in those mortars containing PCM. This behavior is certainly due to the lower mechanical performance measured on the CEM_PCM mortar (as observed in Figure 9). It is likely that, as these mortars are less resistant, they are also more prone to developing microcracks in their matrix as a result of pressures generated in the microstructure due to the expansion of water volume during freezing, despite having lower porosity (as illustrated in Figure 6 and Figure 7). These results confirm the importance of analyzing the influence of PCMs on mortars, even after exposure to extreme thermal cycles. Mass loss in PCM-containing mortars can affect the durability and integrity of these materials in applications involving significant temperature fluctuations.

It is, therefore, fundamental in the construction sector to know these characteristics for an adequate choice of materials suitable for different environmental contexts. However, it must be highlighted that the CEM_PCM mortar developed in the present study shows limited degradation compared to mortars incorporating microencapsulated PCM, as reported in previous studies [50]. Several studies have also demonstrated the vulnerability to freeze–thaw cycles of cement mortars added with non-encapsulated PCM [51]. The greater sensitivity of mortars to freeze–thaw actions, measured by a greater mass loss of the specimens, was also measured in mortars containing superabsorbent polymers [71] and in those that incorporate recycled cement powder [72].

Analyzing the mechanical behavior of the mortars after undergoing several freeze–thaw cycles (i.e., 56 cycles) revealed a decrease in their compressive strength for both the PCM-containing mortars and the reference mortars. However, an interesting point to consider is that the reference mortars experienced a greater reduction in mechanical performance of about 38% compared to the PCM-containing mortars, which saw a decrease of only 18%. This evidence is significant because it shows how the presence of PCM effectively mitigates temperature variations, thereby minimizing the detrimental effects of freeze–thaw cycles [35]. It is likely that the aggregate containing the PCM was not significantly affected by the freeze–thaw cycles thanks to the saturation of the pores of the LS matrix by PEG.

In recent investigations, PCMs have even been used to improve the freeze–thaw resistance of mortars [35] and concrete structures [73], especially by improving their compressive strength. However, PCMs with low transition temperatures, i.e., around 5 °C, are generally selected for this purpose. Therefore, the behavior of PCM-containing mortars with higher phase change temperatures has not been sufficiently investigated. Current literature reports only a few studies focused on the compressive strength of cement mortars incorporating different polymers with high transition temperatures, such as polyurethane [71], acrylic acid, and ammonium acrylate [74]. In these studies, a decrease in compressive strength is observed after the action of the freeze–thaw cycles, with a more significant reduction as the number of cycles increases. However, the addition of these polymers is able to mitigate the effect of freeze–thaw cycles compared to the reference mortars, i.e., those not containing any polymer. Finally, the adhesion tests performed on both mortars under analysis before and after the freeze–thaw cycles showed a decrease in adhesion of approximately 43% for the reference mortar compared to standard conditions, while the mortars containing the aggregates impregnated with PCM showed very brittle behavior, and it was not possible to measure their adhesive strength. The behavior of the CEM_PCM mortar can be justified by excessive fragility resulting from the freezing and thawing cycles of water, as evidenced by the low value of compressive strength (Figure 12) and the greater mass loss of the samples subjected to this severe aging (Figure 13).

## 6. Conclusions

In this study, the physical and mechanical behavior of cement mortars containing or not aggregates impregnated with PEG-based phase change material was investigated, both in standard conditions or subjected to freeze–thaw cycles. Based on the results obtained, the following conclusions can be drawn:

1. The incorporation of form-stable PEG-based PCM aggregates resulted in a decrease in the water absorption capacity, both by capillarity and by immersion. This was explained by the saturation of the aggregate with the PEG, which constitutes the PCM, thus greatly reducing the porosity of the LS. In addition, even the lower water/binder dosage used in these mortars contributes to obtaining a more compact and less porous mortar, further justifying the lower water absorption capacity found.
2. The mechanical performance of the cement mortar, evaluated by the compressive strength and adhesion developed on a ceramic substrate, was reduced by the presence of the aggregates containing the PEG-based PCM. This behavior was justified by a delay in the cement hydration process due to the PCM, which may also be present on the surface of the aggregate particles, and by the lower bond strength that may have developed between the PCM-impregnated aggregate and the cement paste. Nonetheless, even the mortar containing PEG-based PCM shows adequate behavior for applications in construction, according to current European standards.
3. Freeze–thaw damage was more significant for aggregate-based mortars containing PCM, as evidenced by greater mass loss and a severe decrease in compressive and adhesive strength. However, the decrease in compressive strength measured for the mortar containing PEG-based PCM after the freeze–thaw cycles was lower than that observed for the reference mortar. This result may reveal a promising behavior of mortars containing PCM that is different from that expected, although the transition temperature of the PEG-based PCM is higher than that reported for other PCMs, which aim to increase the durability of mortars subject to freeze–thaw cycles.

Based on the findings of this research, several directions for further research can be suggested. One avenue could be the investigation of the optimal amount of PEG–PCM to incorporate into the mortar to balance mechanical properties and, consequently, enhance the adhesion capacity, which could help in fine-tuning the material for specific applications. Additionally, conducting long-term durability studies under various environmental conditions, including wet–dry cycles and thermal aging tests, will provide comprehension into the performance and longevity of the PEG–PCM-modified mortars. Assessing the environmental impact and sustainability of using PEG–PCM in construction materials is also crucial. Life cycle analysis (LCA) can be used to evaluate the ecological footprint and potential benefits in terms of energy savings and reduced emissions. Finally, performing a cost-benefit analysis to determine the economic feasibility of using PEG–PCM-modified mortars in various construction scenarios would help understand the trade-offs between initial costs and long-term benefits.

## Figures and Tables

**Table 1:** Mortars formulation (kg/m^3^).

Composition	Cement	Lecce Stone	Lecce Stone with PEG	Superplasticizer	Lecce Stone Water Saturation	Water/Binder
CEM_REF	1000	772	0	20	194	0.39
CEM_PCM	1000	0	1307	20	0	0.30

**Table 2:** Program of the measurements of the freeze–thaw tests.

Measurement	Cycle (Days)	Time (Hours)
1	0	0
2	1	24
3	2	48
4	3	72
5	6	144
6	8	192
7	13	312
8	20	480
9	27	648
10	41	984
11	56	1344

## Data Availability

The original contributions presented in the study are included in the article, further inquiries can be directed to the corresponding author.
